# Biogeochemistry Goes Viral: towards a Multifaceted Approach To Study Viruses and Biogeochemical Cycling

**DOI:** 10.1128/mSystems.01138-21

**Published:** 2021-10-12

**Authors:** Patricia Q. Tran, Karthik Anantharaman

**Affiliations:** a Department of Bacteriology, University of Wisconsin—Madison, Madison, Wisconsin, USA; b Department of Integrative Biology, University of Wisconsin—Madison, Madison, Wisconsin, USA

**Keywords:** bacteriophages, biogeochemistry, microbial ecology, viruses

## Abstract

Viruses are ubiquitous on Earth and are keystone components of environments, ecosystems, and human health. Yet, viruses remain poorly studied because most cannot be isolated in a laboratory. In the field of biogeochemistry, which aims to understand the interactions between biology, geology, and chemistry, there is progress to be made in understanding the different roles played by viruses in nutrient cycling, food webs, and elemental transformations. In this commentary, we outline current microbial ecology frameworks for understanding biogeochemical cycling in aquatic ecosystems. Next, we review some existing experimental and computational techniques that are enabling us to study the role of viruses in biogeochemical cycling, using examples from aquatic environments. Finally, we provide a conceptual model that balances limitations of computational tools when combined with biogeochemistry and ecological data. We envision meeting the grand challenge of understanding how viruses impact biogeochemical cycling by using a multifaceted approach to viral ecology.

## COMMENTARY

## THE IMPORTANCE OF VIRUSES IN AQUATIC BIOGEOCHEMISTRY

Microbial communities are central to biogeochemical cycling, as observed in marine (1), soil (2), and freshwater (3) environments. Over the past decades, technological advances have led to the increase of genomic sequencing, resulting in discoveries about the roles of microbes, particularly bacteria and archaea. However, few studies in aquatic microbial ecology transcend the domains of life to the realm of viruses. This lack of understanding of viruses prevents their inclusion in next-generation models that are being used to inform long-term predictions of metabolism, ecosystems, and biogeochemistry.

**FIG 1:**
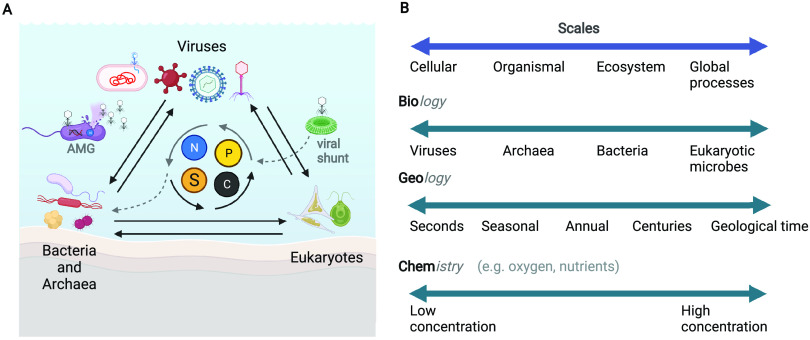
(A) Complex microbial communities made up of viruses, bacteria, archaea, and eukaryotes interact with each other and their environment through mechanisms such as predation and competition for resources. Two specific examples are shown including an example of an auxiliary metabolic gene (AMG)-containing phage that acts on the metabolism of a bacterium to produce more viral progeny (left) and an example of a virus infecting a phytoplankton to influence C, N, P, and S availability, via the viral shunt (right). (B) Different levels of organization contribute to a holistic understanding of ecology and are associated with challenges of studying viruses. Each of the biology, geology, and chemistry components can be studied across a range of scales, from cellular to global processes. The figure was created with BioRender.com.

Most studies either focus on bacteria, archaea, or viruses individually. When combined, studies can explain how sudden shifts in biogeochemical processes in otherwise stable communities are driven by viruses (4). Microorganisms form complex communities that interact with each other through predation mechanisms such as cell lysis, grazing, and competition for resources (Fig. 1A). Therefore, studying how all these drivers interact with each other may provide a mechanistic understanding that goes beyond descriptive ecology.

Viral ecology studies have demonstrated that viral roles in ecosystems cannot be ignored. For example, lytic viruses can target microbes, release carbon that fuels the microbial food web (the viral shunt) (5), and have direct effects on the microbial community composition (6). Additionally, viruses encoding auxiliary metabolic genes (AMGs) can manipulate their hosts and impact microbial metabolism and processes such as carbon, nitrogen, sulfur, and iron cycling (7). These biogeochemical pathways are often tightly associated with environmental conditions such as oxyclines or chemoclines in aquatic ecosystems (Fig. 1B). Most viral genomic studies that specifically address biogeochemical pathways have assessed marine environments. Other aquatic environments, including inland lakes, coastal regions, streams, and rivers, also have dynamic spatiotemporal patterns that are related to microbial (bacterial, archaeal, eukaryotic) roles in biogeochemical cycling but remain understudied in the context of viruses. Evidence points to similarly prominent viral communities in ecosystems such as lakes, where AMG-containing viruses are potentially involved in biogeochemical cycling (4, 8–10). With growing evidence of viral roles in biogeochemical cycling, obtaining a more holistic understanding of functional roles, interactions, and effects of these communities can be achieved by bridging across the bacterium-archaeon-eukaryote-virus boundaries.

## TECHNIQUES TO STUDY THE ROLES OF ENVIRONMENTAL VIRUSES

Experimental and laboratory techniques exist and provide an initial set of tools to begin integrating different scales of biology (Fig. 1). Some methods rely on the ability to culture viruses with their host, whereas others can be performed without a cultivated host. Enumeration of viruses by phage plaque assays show that virus counts vary within an aquatic ecosystem (11). In a global analysis of virus morphology in the oceans, researchers used microscopy to observe that nontailed viruses dominated surface ocean microbial communities (12). By incorporating ecological context, follow-up studies have showed that nontailed viruses in marine environments are a major predation mechanism on bacteria (13). Yet, most viruses studied in culture are tailed, thereby showing the importance of both cultivation-based and cultivation-independent lines of evidence for understanding ecological relevance. Dilution to extinction, another laboratory method, involves filtering water, followed by enrichment, purification, and isolation to finally obtain a virus-host system (14). Model host-virus systems are useful to explore targeted biogeochemical pathways and host-virus interactions since the controlled environment provides higher reproducibility. For example, carbon regeneration could be addressed by changing the abundance of viruses and measuring the host growth rate and biomass over time. Similarly, a host known to be involved in denitrification can be measurably impaired or improved upon the addition of a virus that targets it, by tracking host, viral, and chemical characteristics over time.

One step toward a more holistic understanding of biogeochemical processes in ecosystems is to move beyond studying model organisms to learn about other components of an ecosystem. Additionally, biogeochemistry relies on biology, geology, and chemistry, all of which have various techniques that can help understand the overall impact of viral ecology. Whereas there is a generalized recognition of the need to study uncultured microorganisms (archaea, bacteria, eukaryotes) to understand ecosystem processes, this concept is not as common in the field of virology. Since viruses are dependent on a host for cultivation and most microorganisms in nature cannot be cultivated, few environmentally relevant viruses have been cultured thus far.

To circumvent the limitations of culture-dependent viral ecology, the ongoing development of computational techniques that address the interpretative challenges of viral “omics” data will facilitate environmental virus analysis in complex environmental ecosystems. In the past years, the field of microbial metagenomics (mostly bacteria and archaea) has seen a shift from bulk read-based metagenome characterization toward functional understanding at the scale of metagenome-assembled genomes, and even at strain-level understanding of evolutionary processes and ecological patterns. The improved ability to leverage information from metagenomics is in part due to computational advances like high-throughput sequence processing, genome binning, improved algorithmic efficiency, and standardization of data. Such computational advances may be possible in the future for viral omics. Viral genomic tools are being written, tested, compared, and used to gain ecological insights (15–17), and information is becoming standardized (18). In time, these tools will facilitate a better understanding of viruses and their complex biogeochemical interactions.

Transcending laboratory-only and genomics-only boundaries can lead to novel methods for studying viral ecology that take advantage of both strengths. Single-cell viral tagging and sequencing, analogous in some ways to single-cell genome sequencing of bacteria, rely on tagging viruses with fluorescent dyes and then cell sorting and sequencing to identify actual host-virus interactions without the need for culturing. Single-cell viral tagging and sequencing were developed for the human gut (19). Another technique, epicPCR, consists of linking phylogenetic genes to functional genes, and then uses sequencing to obtain high-throughput ecologically relevant information about cells, such as their roles in sulfate reduction (20). This technique is powerful in identifying virus-host interactions, especially for when viruses are not represented in databases. EpicPCR has been adapted to study virus-host interactions without cultivation in estuarine environments, and differences have been observed in viral lifestyles and strategies between viruses with narrow and broad host ranges in the environment (21). Overall, these examples of technological advances which combine the strengths of laboratory and computation (sequencing and its interpretation) could be applied to viral biogeochemical cycling in aquatic environments. For example, we imagine collecting a time series of microbial samples in an environment that is subject to environmental change (either biological or abiotic) using any of the aforementioned methods to sequence and study virus-host interactions. This would typically be followed by genomic investigation of viruses and hosts and interpretation of their involvement in carbon, nitrogen, or sulfur biogeochemical cycling over time, while being certain that the physical interaction between the viruses and their hosts exists. All these techniques highlight the future of viral ecology and the potential for their application across aquatic ecosystems.

## LOOKING FORWARD: COMBINING MULTIFACETED APPROACHES IS IMPORTANT TO OBTAIN A HOLISTIC UNDERSTANDING OF ECOSYSTEM ECOLOGY

**FIG 2:**
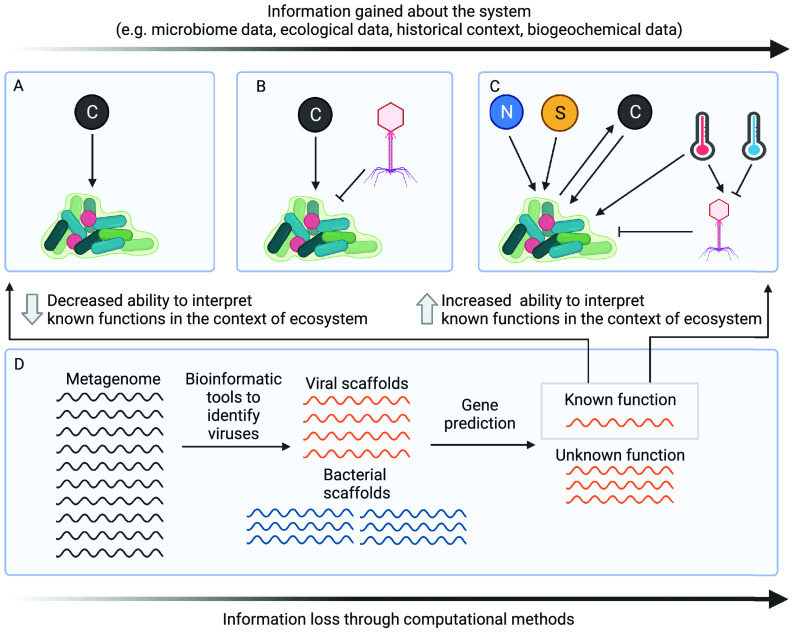
Conceptual framework for maximizing information about viral ecology and biogeochemistry in nature. Along the axis at the top of the figure are ways to gain more information about a system. (A) Example showing the positive feedback of carbon on bacterial growth. (B) Addition of viruses increases complexity over that shown in panel A. (C) Further adding detailed biogeochemical and environmental metadata such as carbon, nitrogen, sulfur, and temperature can relate complex environmental conditions to ecology but increases complexity over the complexity shown in panels A and B. Examples of positive (arrow tip) and negative (inhibitor tip) interaction are shown. (D) Loss of information across various steps of computational analyses in viral ecology. The loss of information from computational analysis can be balanced by information gained from biogeochemical and environmental metadata. The figure was created with BioRender.com.

The amount of genomic data generated has exponentially increased in recent years, and their interpretation benefits from a thorough understanding of the historical and ecological context and of future challenges that the ecosystem may encounter (Fig. 2). We believe that the ability to interpret viral ecology data, particularly omics based, will be facilitated by collecting metadata and contextualizing the study system. For example, one could study the impact of carbon on bacterial growth at various resolutions ranging from simple studies focused on positive feedback at an organismal or community level (Fig. 2A) to increasing complexity of interactions (Fig. 2B and C). Moving toward more integrative studies, the incorporation of multiple species, multiple realms of life, and comprehensive metadata about biogeochemistry and the environment will allow us to determine complex positive and negative feedback in the system (Fig. 2). Specifically in the case of viral ecology, we suggest that standard virus sampling methods be coupled with detailed metadata collection of biogeochemistry and microbial communities (bacteria, archaea, and eukaryotes), which could greatly increase the ability to interpret and synthesize results.

Figure 2D demonstrates how computational techniques and their results, while offering a glimpse into viral ecology, remain challenging to interpret. In the simplified example, a metagenome generated from a given sample is used as a starting point to computationally identify viruses. Along each step of the pipeline, context is lost because a relatively low percentage of viruses are identified, of which most viral genomes are partial, and even fewer of the identified viruses have an identified ecological function or role. The analysis of viral genomics can be challenging on its own, especially where viral bioinformatic methods remain in constant development and have their own shortcomings. Given the same genomic data set and outcome, the ability to interpret ecological functions is significantly increased with the availability of comprehensive metadata and biogeochemical data (Fig. 2C and D) compared to without this data (Fig. 2A).

Finally, we envision that full integration of viral ecology into measurable and predictable outcomes would involve its integration into biogeochemical and ecosystem models. Substantial efforts have been made to integrate metagenomic and metatranscriptomic data of microorganisms (bacteria and archaea) in predicting biogeochemical processes such as carbon, nitrogen, and sulfur cycling across redox gradients (22, 23). Realistically, it has taken over a decade of work for the field of (bacterial and archaeal) metagenomics to move on from descriptive studies of biodiversity toward mechanistic and predictive models that integrate multiple lines of experimental and genomic evidence. Even so, these integrative studies are not the norm. While challenges and opportunities in viral ecology will involve overcoming resource limitations and cross-disciplinary learning curves, we envision gaining the ability to closely couple viral ecology and biogeochemistry through these multifaceted efforts.
